# Mechanism of health literacy impact on self-management behaviors in patients with chronic disease: a self-efficacy mediated model moderated by disease duration

**DOI:** 10.3389/fpubh.2025.1673723

**Published:** 2025-11-10

**Authors:** Shichen Chen, Yike Wang, Wenjing Liu, Die Zhang, Jinghui Zhu, Guilin Liu

**Affiliations:** School of Medical Humanities and Management, Wenzhou Medical University, Wenzhou, Zhejiang, China

**Keywords:** health literacy, disease course, self-efficacy, self-management behavior, moderated mediation mode, chronic diseases

## Abstract

**Introduction:**

Self-management behaviors are vital in chronic disease prevention and management, with self-efficacy acting as a key mediator between health literacy and these behaviors. However, disease duration may amplify or attenuate health literacy's impact on self-efficacy—either through experiential learning or management fatigue—requiring empirical validation of its moderating role. This study thus applied a moderated mediation framework to investigate how multidimensional health literacy influences self-management via self-efficacy, and whether disease duration moderates the health literacy–self-efficacy pathway, aiming to clarify efficacy belief dynamics in long-term adaptation.

**Methods:**

A cross-sectional study of 601 patients with chronic disease in Wenzhou's Ouhai District assessed health literacy, self-efficacy, and self-management. Using Hayes' PROCESS macro (Model 4 for mediation analysis and Model 7 for moderated mediation analysis, with 5,000 bootstrap iterations).

**Results:**

We found that self-efficacy partially mediated the relationship between health literacy and self-management behaviors (ES = 0.082, 95%CI: 0.055–0.110; 22.601% of total effect). Crucially, disease duration positively moderated the effect of health literacy on self-efficacy (*B* = 0.014, *p* < 0.05, 95%CI: 0.002–0.026), strengthening the indirect effect of health literacy on self-management through self-efficacy as duration increased.

**Discussion:**

These findings demonstrate that self-efficacy mediates the health literacy–self-management link, while disease duration enhances health literacy's effect on self-efficacy, supporting stage-specific precision interventions.

## Background

Chronic diseases have emerged as a primary challenge in the field of public health (1). Central to addressing this challenge are patient self-management behaviors, which refer to a set of skills and practices that individuals acquire and apply to manage their daily health. These encompass medication adherence, physical activity, symptom management, proactive information-seeking, and effective communication with healthcare providers (2). It has been widely demonstrated that effective self-management behaviors are beneficial for slowing the progression of the disease (3), improving the quality of life (4), and prevention of complications (5). Effective self-management behaviors are heavily reliant on patients' capacity to comprehend and process health information (health literacy) (6) and their belief in behavioral regulation (self-efficacy) (7).

Health literacy significantly influences patients' self-management behaviors (8). Specifically, studies have shown that low levels of health literacy are associated with irrational life behaviors (9, 10), poor treatment adherence (11, 12), ineffective disease self-management (13). However, traditional studies often oversimplify health literacy as a unidimensional functional competence, focusing solely on patients' literacy levels and their understanding of basic information such as medical instructions and medication labels (14). This simplistic approach fails to explain behavioral disparities in disease management among patients with similar educational backgrounds and inadequately addresses the complex demands of chronic disease management, including social interactions, proactive decision-making, and resource coordination (15). To address this limitation, Nutbeam proposed a multidimensional health literacy model (16). This framework categorizes health literacy into three tiers: functional health literacy (basic knowledge and skills in healthcare activities), communicative health literacy (information exchange and dissemination in social interactions), and critical health literacy (analysis and evaluation of health information), thereby providing a more comprehensive theoretical foundation for analyzing patient behaviors (17). Subsequently, scholars introduced distributed health literacy (18), emphasizing individuals' capacity to mobilize social network resources for collaborative health problem-solving (19), which may mitigate the adverse effects of low individual-level health literacy (20).

Rooted in social cognitive theory, self-efficacy is defined as an individual's confidence in their ability to organize and execute courses of action required to accomplish specific tasks and attain desired outcomes (21). The theory posits that the initiation of health behaviors typically undergoes a chain reaction of “cognitive capacity → efficacy belief → behavioral practice” (21). Specifically, health literacy empowers patients with chronic disease through multidimensional competencies—including understanding medical instructions, communicating symptoms, evaluating risks, and utilizing resources (20, 22) (cognitive capacity)—which strengthen their belief in disease control (23) (efficacy belief), thereby enabling them to maintain adherence during health crises (24), and implement improved self-management behaviors (25) (behavioral practice). This “capacity-belief-behavior” chain transformation suggests that self-efficacy may serve as a critical mediating variable through which health literacy influences self-management behaviors (26). For instance, studies on diabetic populations demonstrate that enhanced health literacy significantly elevates self-efficacy levels, ultimately leading to effective glycemic control (27, 28). Scholars Paasche-Orlow and Wolf further corroborate the mediating role of self-efficacy in linking health literacy to self-management behaviors (29).

Although prior studies have established health literacy as a key antecedent predictor of self-management behaviors (30) and self-efficacy as a pivotal mediator linking health literacy to self-management behaviors (26), conflicting evidence persists regarding the outcomes of health literacy's influence on self-management behaviors through self-efficacy, with current findings primarily derived from diabetic populations. For instance, a study investigating health literacy, self-efficacy, self-care behaviors, and glycemic control among older adults with type 2 diabetes demonstrated that those with higher health literacy levels developed heightened self-efficacy through repeated management of glycemic fluctuations, thereby enabling better glycemic control and greater willingness to engage in proactive health management behaviors (31). Conversely, Lee et al., employing structural equation modeling to analyze relationships between health literacy, self-efficacy, and self-care in patients with type 2 diabetes (32), revealed weaker efficacy beliefs in self-care among patients with high-health-literacy, which subsequently led to difficulty maintaining stable self-management adherence (33). This inconsistency, though predominantly observed in diabetes, underscores the necessity to further elucidate the intrinsic pathway mechanisms through which health literacy affects self-management behaviors via self-efficacy across diverse chronic disease contexts.

The Disease Adaptation Theory (34) offers a novel perspective: health literacy and self-efficacy in patients with chronic disease are not static attributes but rather undergo dynamic interaction and mutual influence throughout disease progression. Some scholars posit that patients with longer disease duration may accumulate more disease management experience through prolonged adaptation processes, thereby amplifying health literacy's facilitative effect on self-efficacy (31). However, counterarguments suggest that extended disease duration may engender fatigue or psychological burdens from sustained disease management, potentially undermining self-efficacy (35). Even with high health literacy, patients might experience compromised self-management effectiveness due to treatment fatigue or wavering health beliefs (36). These findings collectively indicate that disease duration functions not merely as a temporal indicator of pathological progression but as a critical moderating variable influencing the relationships among health literacy, self-efficacy, and self-management behaviors—potentially regulating both the magnitude and directionality of health literacy's impact on self-efficacy.

This study constructs a multidimensional integrated health literacy assessment framework comprising functional, communicative, critical, and distributed components, forming a competency chain of “foundational execution → information exchange → autonomous decision-making → systemic support,” (20, 37–40) with its evaluation outcomes being better aligned with the complex demands of chronic disease management (7). While existing research has established self-efficacy as a key mediator linking health literacy to self-management behaviors, its mediating role within a multidimensional health literacy framework requires further verification. Concurrently, prior studies have yet to adequately elucidate the dynamic moderating effects of disease progression on this mediating pathway.

Therefore, this study aims to address two key research questions through a moderated mediation model: (i) whether multidimensional health literacy influences self-management behaviors through self-efficacy as a mediator, and (ii) whether disease duration exerts moderating effects between health literacy and self-efficacy, including the directionality and magnitude of such effects. By implementing this framework, the research innovatively examines the mechanism of self-efficacy's mediating role within multidimensional health literacy constructs, overcoming the limitations of traditional health literacy studies that focus solely on individual competence. Furthermore, it elucidates the temporal dynamics of disease duration in regulating the “capacity-belief” transformation pathway, thereby offering a novel paradigm for deconstructing adaptive processes in chronic disease management and developing phase-specific precision intervention strategies.

## Methods

### Sample and procedure

The study employed a stratified sampling method to select primary healthcare institutions in Ouhai District, Wenzhou City as research sites. Based on financial data from the Ouhai District Statistical Yearbook (2023), subdistrict jurisdictions were categorized into low-, medium-, and high-economic tiers. One subdistrict from each tier was randomly selected through simple random sampling, with their respective community health service centers designated as investigation sites. Sample size calculation using the stratified sampling formula with parameter inputs yielded a theoretical requirement of 609 participants, with 601 valid questionnaires ultimately collected. To ensure representativeness, sample quotas were proportionally allocated across subdistricts according to their resident population sizes.

Prior to data collection, researchers conducted standardized training to ensure quality control, emphasizing: prohibition of interpretive paraphrasing or response guidance during questionnaire administration; mandatory use of neutral language; Establishment of uniform response protocols. Data were gathered through face-to-face interviews following a structured protocol: first, detailed explanations of research objectives, procedures, potential risks/benefits were provided, with written informed consent obtained after confirming participants' comprehension. Post-survey, participants received hygiene products valued at ¥5 as non-coercive compensation, approved by ethics review as non-inducement. The health literacy and other assessment instruments were originally designed as a written self-report instrument; however, to accommodate participants with literacy limitations, the scales were administered orally through verbatim reading of items and response options by trained interviewers, ensuring consistency and minimizing bias. This adaptive approach maintained the integrity of the multidimensional health literacy construct while enabling inclusive participation. All data underwent completeness checks and logical validation before analysis, with the entire investigation completed over 26 days. Inclusion criteria comprised: (i) age ≥45 years (threshold corresponding to epidemiologically significant chronic disease prevalence increases); (ii) clinically confirmed diagnosis of ≥1 chronic condition, verified through a combination of medical records review, physician assessments from the participating primary healthcare institutions, and where applicable, confirmation of long-term regular use of typical chronic disease medications (e.g., antihypertensives, hypoglycemic agents); (iii) voluntary provision of informed consent. Exclusion criteria included: (i) diagnosed psychiatric disorders or cognitive impairments, which were identified through medical records review and assessments by healthcare providers during recruitment; (ii) communication barriers compromising data validity, discerned through initial screening and observational evaluation by trained interviewers, who assessed participants' ability to understand questions and respond coherently during the consent process and interviews.

This study received ethical approval from Wenzhou Medical University Ethics Committee (Approval No.: 2024067), strictly adhering to the Helsinki Declaration and Chinese regulatory requirements.

### Instruments

Data collection was conducted using a standardized questionnaire comprising four sections: sociodemographic and health status information, a health literacy scale, a self-efficacy scale, and a self-management behavior scale. Notably, all scales underwent rigorous translation and cultural adaptation processes to ensure linguistic equivalence and cultural relevance for the target population. An additional file shows this in more detail (see Additional File 1).

The health literacy measurement instrument was developed to address limitations in existing assessment tools characterized by unidimensional constructs (41) or disease-specific orientations (42). Aligned with academic consensus on optimizing health literacy evaluation for chronic disease management (43), this study integrated three theoretical foundations: the cross-cultural adaptation outcomes of China's indigenous HL-14 scale (44), Nutbeam's functional-communicative-critical tripartite framework (16), and distributed health literacy theory (19). The resultant multidimensional scale encompasses four domains: functional (three items), communicative (five items), critical (five items), and distributed health literacy (four items), employing a 5-point Likert scale format. Higher scores indicate superior health literacy levels. The Cronbach's α coefficient for this multidimensional health literacy scale reached 0.917. Confirmatory factor analysis demonstrated that AVE values (0.553–0.785) and CR values (0.830–0.916) satisfied standard thresholds (AVE > 0.5, CR > 0.7), with standardized factor loadings ranging from 0.620 to 0.898. The square roots of AVE values exceeded inter-factor correlations, supporting discriminant validity. The model results showed χ^2^ = 490.711 (df = 113, *p* < 0.001), and other fit indices (CFI = 0.943, NFI = 0.927, RMSEA = 0.075, SRMR = 0.048) were excellent, confirming that the health literacy scale has robust reliability and validity, including high convergent and discriminant validity.

Self-efficacy in chronic disease management was assessed using Lorig et al.'s Self-Efficacy for Managing Chronic Disease 6-Item Scale (45, 46). Grounded in multidimensional theoretical frameworks of chronic disease self-management, this instrument covers critical domains including emotional control, patient-provider communication, symptom management, role functioning, and illness perception adaptation. To enhance cultural appropriateness and reduce respondent burden, the original 10-point Likert scale was adjusted to a 5-point format (47) (1 = “Not confident at all” to 5 = “Completely confident”), with total scores ranging from 6 to 30. Higher scores denote stronger self-efficacy. Despite the simplified rating scale, the adapted self-efficacy measure maintained robust internal consistency, achieving an overall Cronbach's α coefficient of 0.848.

The development of the chronic disease self-management behavior assessment tool integrated the chronic disease self-management behavior theoretical framework (48) with adaptive studies conducted by Chinese scholars within local contexts (49). This process yielded a four-dimensional scale encompassing disease management (three items), lifestyle management (four items), exercise management (two items), and social functioning/interpersonal management (five items). Utilizing a 5-point Likert scale, higher total scores indicate superior self-management behavior levels. Exploratory factor analysis demonstrated satisfactory structural validity (KMO = 0.786, Bartlett's sphericity test *p* < 0.001; four-factor cumulative variance contribution rate = 62.722%), with the scale exhibiting good overall internal consistency (Cronbach's α = 0.804), aligning with multidimensional theories of chronic disease self-management.

Disease duration was assessed through a single self-report item: “How many years have passed since your initial chronic disease diagnosis?” To mitigate recall bias and enhance response feasibility, we implemented an ordinal categorical design with six mutually exclusive intervals: *A*(0–2 years), *B*(2–4 years), *C*(4–6 years), *D*(6–8 years), *E*(8–10 years), *F*(>10 years). Interval boundaries strictly adhered to the left-open-right-closed principle in epidemiological studies to eliminate ambiguity in duration classification. Supported by methodological evidence (50), ordinal coding effectively preserves gradient information and enables parametric analyses when categories demonstrate clear monotonicity with equal spans (2-year intervals in this study). Consequently, categorical variables were converted to continuous numerical values (*A* = 1, *B* = 2,..., *F* = 6), where ascending values directly reflect increasing disease duration.

### Statistical analyses

**Figure 1:**
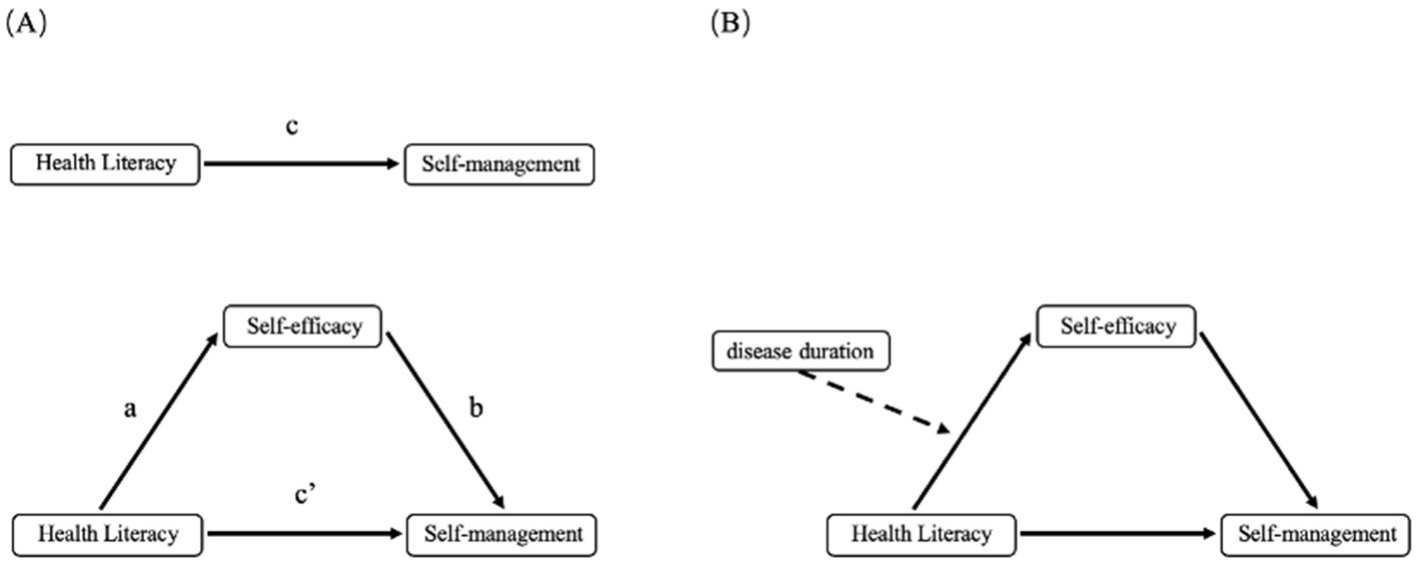
**(A)** The mediating effect of self-efficacy between health literacy and self-management behaviors; **(B)** The moderating effect of disease duration between health literacy and self-efficacy.

The data analysis was conducted using IBM SPSS Statistics version 27. Descriptive statistics (mean, standard deviation, frequency, and percentage) were employed to present demographic characteristics. Independent samples *t*-tests and analysis of variance (ANOVA) were performed to examine differences in sample characteristics regarding self-management behaviors. Spearman correlation analysis was conducted to examine inter-variable correlations. In the conceptual model (Figure 1), path c represents the total effect of the predictor variable health literacy on the outcome variable self-management behavior. This total effect comprises both the direct effect of health literacy on self-management behavior (path c') and the indirect effect mediated through the mediating pathway of self-efficacy (path a^*^b). The study utilized Hayes' PROCESS macro version 4.0.1 for mediation effect and moderated mediation model analyses. A bias-corrected 95% confidence interval (CI) was calculated using 5,000 Bootstrap resamples. First, Model 4 was applied to test the mediating role of self-efficacy between health literacy and self-management behavior. A statistically significant mediation effect was established if the 95% CI for the indirect effect (path a^*^b) excluded zero. Subsequently, Model 7 was employed to examine whether disease duration moderated path a (health literacy → self-efficacy). A significant moderated mediation effect was confirmed if the 95% CI for the interaction term excluded zero. Finally, simple slope analysis was conducted to facilitate interpretation of the interaction effect diagram (Figure 1).

## Results

### Sociodemographic characteristics and self-management

The study included 601 participants with the following demographic characteristics: a gender-balanced composition, 63.6% aged ≥65 years, 84.6% having primary education or below, and occupational profiles predominated by staff and retirees (78.0%). Most participants were married (86.7%), resided in rural areas (56.1%), and reported annual personal incomes ≤ ¥40,000 (68.6%). Regarding disease profiles, 66.1% had a single chronic condition, 37.1% exhibited overweight/obesity, and 43.9% self-rated their health status as “good.” Data analysis demonstrated statistically significant differences in self-management behavior scores across 11 variables including gender, age, and educational attainment (Table 1).

**Table 1:** Description of the socio-economic data (*N* = 601).

**Variables**	***n***	**Percentage**	**Self-management behavior**	
			$\bar{x}\pm s$	***t/F***
**Gender**				
Male	311	51.7	49.88 ± 6.07	−7.602^**^
Female	290	48.3	53.68 ± 6.18	
**Age group (years old)**				
45	70	11.6	53.94 ± 6.09	12.774^**^
55	149	24.8	53.15 ± 6.43	
65	382	63.6	50.75 ± 6.27	
**Education**				
Illiterate	340	56.6	50.44 ± 6.15	12.005^**^
Primary school	168	28.0	52.55 ± 6.32	
Middle school	70	11.6	54.13 ± 6.44	
High school	17	2.8	56.00 ± 4.97	
College or above	6	1.0	60.33 ± 3.56	
**Occupation**				
Professional personnel	5	0.8	61.00 ± 3.54	3.992^**^
Office workers	12	2.0	54.33 ± 6.12	
Company staff	67	11.1	53.63 ± 6.43	
Farmers	42	7.0	50.24 ± 6.90	
Freelancer	46	7.7	52.70 ± 6.39	
Unemployed	25	4.2	50.08 ± 5.31	
Retire	402	66.9	51.38 ± 6.29	
Other	2	0.3	44.50 ± 3.54	
**Marital status**				
Married, cohabiting	508	84.5	52.23 ± 6.14	7.375^**^
Married, not cohabiting	13	2.2	49.15 ± 5.46	
Divorce	5	0.8	46.80 ± 5.36	
Widowed	75	12.5	49.03 ± 7.51	
**Residence**				
City	145	24.1	55.30 ± 5.57	46.731^**^
Urban-rural fringe	119	19.8	52.92 ± 6.45	
Countryside	337	56.1	49.75 ± 5.96	
**Family members**				
1–2	332	55.2	50.79 ± 6.25	7.978^**^
3–5	221	36.8	52.80 ± 6.40	
6–9	48	8.0	53.13 ± 6.62	
**Personal annual income (RMB)**				
< 40,000	412	68.6	51.20 ± 6.35	4.720^**^
40,000	77	12.8	52.57 ± 6.12	
60,000	38	6.3	55.39 ± 6.76	
80,000	37	6.2	52.95 ± 6.17	
100,000	24	4.0	52.75 ± 5.10	
≥150,000	2	0.3	51.50 ± 10.61	
Don't know or refuse to answer	11	1.8	46.00 ± 5.55	
**Annual household income (RMB)**				
< 50,000	151	25.1	49.36 ± 6.19	7.490^**^
50,000	163	27.1	51.21 ± 5.26	
100,000	57	9.5	51.88 ± 7.17	
150,000	71	11.8	54.72 ± 6.05	
200,000	60	10.0	54.30 ± 6.10	
250,000	28	4.7	53.11 ± 5.78	
≥300,000	11	1.8	53.36 ± 6.62	
Don't know or refuse to answer	60	10.0	51.75 ± 7.65	
**Types of chronic diseases**				
1	397	66.1	51.63 ± 6.48	4.846^**^
2	199	33.1	52.09 ± 6.16	
3	5	0.8	43.20 ± 4.76	
**Body mass index (BMI)**				
< 18.5	22	3.7	53.36 ± 6.81	1.367
18.5	356	59.2	51.99 ± 6.61	
24	199	33.1	51.12 ± 6.15	
≥28	24	4.0	51.04 ± 4.68	
**Self-rated health**				
Very bad	3	0.5	52.33 ± 7.37	3.928^**^
Bad	118	19.7	50.24 ± 7.62	
Neutral	216	25.9	51.30 ± 6.11	
Good	247	41.1	52.57 ± 5.88	
Very good	17	2.8	54.76 ± 6.06	

^*^p < 0.05; ^**^p < 0.01.

### Bivariate correlations among the variables

Correlation analysis revealed significant positive associations between health literacy and both self-efficacy (*r* = 0.489, *p* < 0.01) and self-management behaviors (*r* = 0.542, *p* < 0.01). Additionally, self-efficacy demonstrated a statistically significant positive correlation with self-management behaviors (*r* = 0.456, *p* < 0.01; Table 2).

**Table 2:** Statistical description and related analysis results.

**Variables**	** $\bar{x}\pm s$ **	**HL**	**SE**	**SM**
HL	49.925 ± 10.834	1		
SE	21.817 ± 3.309	0.489^**^	1	
SM	51.715 ± 6.410	0.542^**^	0.456^**^	1

^*^p < 0.05; ^**^p < 0.01.

HL, health literacy; SE, self-efficacy; SM, self-management.

### Mediation analyses

The mediating role of self-efficacy between health literacy and self-management behaviors was examined using Model 4 from Hayes' PROCESS macro. After adjusting for 11 covariates including gender, age, and educational attainment, regression analysis revealed a significant total effect of health literacy on self-management behaviors (Path c: *B* = 0.362, *p* < 0.01). The significant coefficient for Path a (*B* = 0.169, *p* < 0.01) indicated a strong association between health literacy and self-efficacy, while the significant Path b coefficient (*B* = 0.479, *p* < 0.01) confirmed the predictive relationship between self-efficacy and self-management behaviors (Table 3).

**Table 3:** Mediation analysis.

**Path**	**Predictor variable**	**Outcome variable**	***B***	***t***	***R^2^***	***F***
c	HL	SM	0.362	14.255^**^	0.510	50.983
a	HL	SE	0.169	11.388^**^	0.368	28.502
c'	HL	SM	0.280	10.417^**^	0.548	54.840
b	SE	SM	0.479	7.096^**^		

^*^p < 0.05; ^**^p < 0.01.

HL, health literacy; SE, self-efficacy; SM, self-management.

Bootstrap-based mediation analysis revealed dual-pathway mechanisms underlying health literacy's influence on self-management behaviors: an indirect pathway mediated through self-efficacy [path a^*^b = 0.082, 95% CI (0.055, 0.110)], accounting for 22.601% of the total effect; and a persistent direct effect [path c' = 0.280, 95% CI (0.228, 0.333)], representing 77.399% of the total effect (Table 4). These results confirm that self-efficacy serves as a statistically significant partial mediator in the relationship between health literacy and self-management behaviors.

**Table 4:** Bootstrap analysis for total, direct, and mediation effects.

**Effect type**	**ES**	**Boot SE**	**Boot 95% CI**		**RE%**
			**LL**	**UL**	
Total effect	0.362	0.025	0.312	0.412	
Direct effect	0.280	0.027	0.228	0.333	77.399%
Indirect effect	0.082	0.014	0.055	0.110	22.601%

ES, effect size; SE, standard error; CI, confidence interval; RE, relative mediation effect.

### Moderated mediation analyses

**Figure 2:**
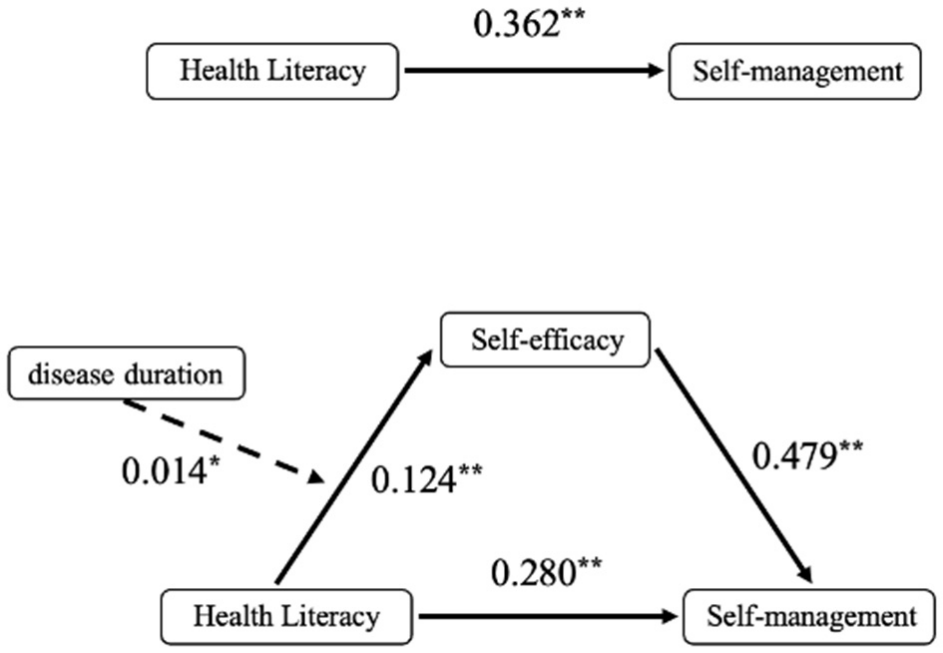
The final moderated mediation model. *p < 0.05; **p < 0.01.

The moderating role of disease duration in the relationship between health literacy and self-efficacy was tested using Model 7 from Hayes' PROCESS macro. Regression analyses adjusted for 11 covariates (including gender, age, and educational attainment) demonstrated (Table 5) that health literacy retained a significant positive effect on self-efficacy after incorporating disease duration as a moderator in Path a (*B* = 0.124, *p* < 0.01). Additionally, a statistically significant positive predictive effect of the health literacy × disease duration interaction term on self-efficacy was observed (*B* = 0.014, *p* < 0.05), demonstrating moderated mediation where disease duration amplifies health literacy's influence on self-efficacy (Figure 2).

**Table 5:** Moderated mediation analysis.

**Predictor variable**	**Outcome variable**	***B***	***t***	**LLCI**	**ULCI**
HL	SE	0.124	5.000^**^	0.075	0.172
HL^*^DD	SE	0.014	2.316^*^	0.002	0.026
HL	SM	0.280	10.417^**^	0.228	0.333
SE	SM	0.479	7.096^**^	0.347	0.612

^*^p < 0.05; ^**^p < 0.01.

HL, health literacy; SE, self-efficacy; SM, self-management; DD, disease duration;CI, confidence interval.

The study further calculated self-efficacy mediation effect sizes and their 95% Bootstrap confidence intervals using Mean ±1 standard deviation (SD) of disease duration as thresholds (Table 6). Results demonstrated that when disease duration was at −1 SD below the mean, the mediation effect of self-efficacy reached 0.070 [95% CI (0.046, 0.098)]. As disease duration increased to +1 SD above the mean, this mediating effect significantly intensified to 0.094 [95% CI (0.063, 0.130)]. Concurrently, the moderated mediation index yielded a statistically significant value [Index = 0.007, 95% CI (0.228, 0.333)] excluding zero, confirming the significance of the moderated mediation effect.

**Table 6:** The mediating effect of self-efficacy on different levels of disease duration.

**DD**	**Mediating effect size/Index**	**Boot SE**	**Boot LLCI**	**Boot ULCI**
Mean-1SD	0.070	0.013	0.046	0.098
Mean	0.082	0.014	0.056	0.111
Mean+1SD	0.094	0.017	0.063	0.130
Index of moderated mediation	0.007	0.003	0.001	0.014

1SD, one standard deviation; DD, disease duration; SE, standard error; CI, confidence interval.

**Figure 3:**
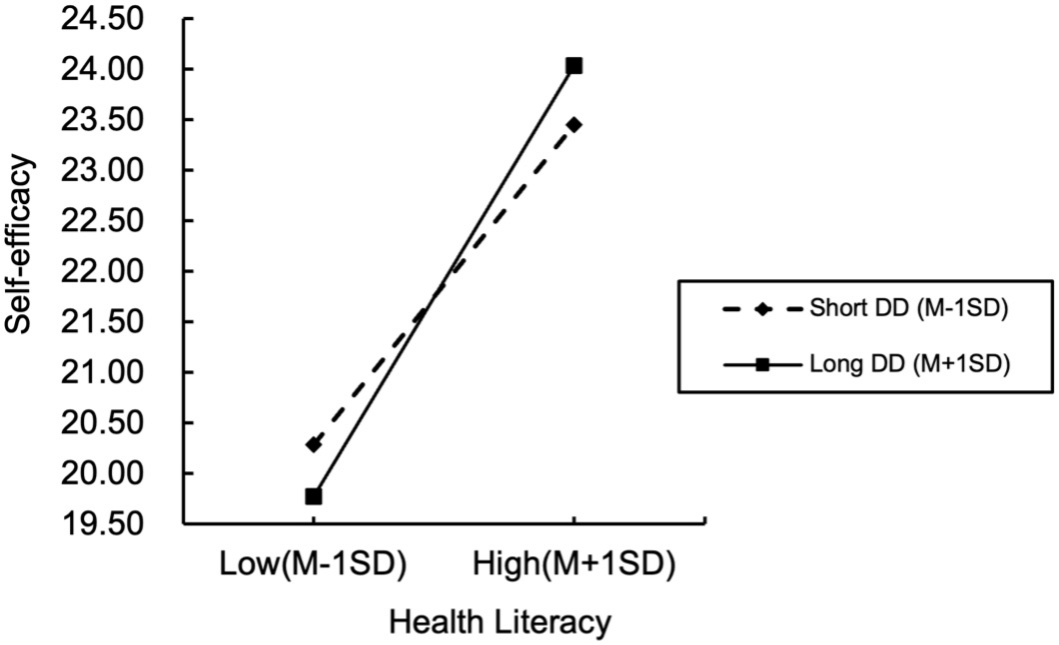
Moderating effects of disease duration on the relationship between health literacy and self-efficacy. DD, disease duration; SD, standard deviation.

Figure 3 visually demonstrates differential effects of health literacy on self-efficacy across disease duration strata. The horizontal axis represents health literacy levels, while the vertical axis indicates self-efficacy scores. Dashed and solid lines delineate relationships between health literacy and self-efficacy for shorter disease duration (Mean −1SD) and longer disease duration (Mean +1SD), respectively.

As illustrated in Figure 3, positive associations between health literacy and self-efficacy persist across all disease duration groups, albeit with a steeper slope observed among patients with longer disease duration. Quantitatively, each unit increase in health literacy corresponded to a weaker incremental effect on self-efficacy for patients with shorter disease duration, compared to a significantly stronger effect for those with longer disease duration. This gradient pattern robustly validates disease duration's moderating role in the health literacy-self-efficacy relationship, demonstrating amplified health literacy benefits on self-efficacy with prolonged disease duration.

## Discussion

This study revealed three key findings with significant theoretical and practical implications. First, health literacy exerted both direct and indirect effects on self-management behaviors among patients with chronic diseases, demonstrating its foundational role in enabling effective disease management. Second, self-efficacy partially mediated this relationship, indicating that confidence in disease control serves as a critical psychological bridge transforming cognitive capability into behavioral execution. Third, disease duration positively moderated the effect of health literacy on self-efficacy, suggesting that prolonged disease experience strengthens the conversion of health literacy into stable efficacy beliefs. Collectively, these findings highlight the importance of integrating health literacy enhancement with efficacy belief reinforcement and considering disease duration in designing stage-specific interventions for chronic disease management.

### Health literacy drives self-management behaviors in patients with chronic diseases

The study identified a statistically significant direct effect of health literacy on self-management behaviors in patients with chronic disease (*B* = 0.280, *p* < 0.01), accounting for 77.399% of the total effect. This demonstrates that enhancing health literacy directly facilitates effective self-management practices, reaffirming its critical role in chronic disease management (6). Aligned with the “cognitive-behavioral” pathway in social cognitive theory (35), health literacy—as the core cognitive component—directly determines the efficiency and sustainability of behavioral implementation. Specifically, patients with advanced health literacy can promptly translate their competencies into concrete actions such as medication adherence and dietary control. This reduces decision-making hesitancy and behavioral procrastination while improving behavioral coherence and precision (17), establishing a direct “cognition-to-behavior” linkage.

Furthermore, through mediation modeling, this research confirms self-efficacy's partial mediating role (indirect effect proportion: 22.601%) in the health literacy → self-management behavior pathway. Mechanistically, health literacy enhances self-efficacy (Path a: *B* = 0.169, *p* < 0.01), which subsequently promotes superior self-management practices (Path b: *B* = 0.479, *p* < 0.01), forming a “cognitive capacity → efficacy belief → behavioral practice” transmission chain. These findings elucidate the psychological mechanism underlying this relationship: patients' ability to acquire, comprehend, and apply health information strengthens their confidence in disease management capabilities, thereby driving sustained behavioral engagement (24). Self-efficacy's bridging role offers crucial implications for intervention strategies: health literacy enhancement must be paralleled with efficacy belief reinforcement. Practical approaches include leveraging success experience feedback and social support systems (23) to transform health literacy into enduring behavioral motivation. The incomplete mediation effect further highlights theoretical opportunities to explore complementary roles of other psychological or contextual variables—such as illness perceptions, social support networks, and healthcare accessibility (23, 51, 52). Future investigations should systematically examine these factors to fully unravel the complex mechanisms through which health literacy shapes self-management behaviors.

### Disease duration positively moderates the impact of health literacy on self-efficacy

The study further revealed that disease duration significantly and positively moderated the effect intensity of health literacy on self-efficacy (*B* = 0.014, *p* < 0.05), demonstrating its critical regulatory role in the dynamic process through which health literacy transforms into efficacy beliefs. To clarify the relational structure, it is essential to note that health literacy, self-efficacy, and disease duration are interconnected within a progressive behavioral adaptation framework. Health literacy provides the cognitive foundation enabling patients to acquire and interpret medical information; self-efficacy represents the psychological confidence to translate this knowledge into consistent self-management behaviors; and disease duration serves as a temporal context that strengthens this conversion process through experiential accumulation. Specifically, patients with longer disease durations exhibited greater capacity to convert health literacy into stable self-efficacy (Figure 3). This finding aligns closely with the Disease Adaptation Theory (34), which emphasizes that individuals' psychological and behavioral adjustments to chronic disease evolve dynamically over time through continuous learning and adaptation. This theory provides a coherent framework for understanding the observed divergence between short- and long-duration patients. In the early stages of adaptation, corresponding to shorter disease duration, patients are often overwhelmed by information and immediate demand. Here, health literacy is primarily utilized for foundational tasks like comprehending instructions and seeking viable management strategies (13), operating more as a reactive tool than being integrated into a stable system of self-belief. Conversely, as postulated by the theory, prolonged duration facilitates a process of experiential learning and cognitive reframing. Patients with longer disease durations accumulate successful management experiences and, through repeated practice, progressively transform their health literacy into internalized, reliable strategies. This process, central to successful adaptation, fosters a consolidation of confidence—their health literacy becomes not just knowledge but a proven asset, thereby strengthening their self-efficacy and enabling more effective self-management behaviors (23). Although the moderating effect size is relatively small compared to previous studies, its statistical significance and directional consistency indicate that disease duration does strengthen the relationship between health literacy and self-efficacy, characterized by a gradual enhancement rather than an abrupt shift. Small incremental changes may accumulate over time into significant behavioral improvements. This modest effect reflects the multifactorial nature of behavioral adaptation in chronic disease patients, where disease duration acts more as a contextual stabilizer than a deterministic variable. The small effect size may be attributed to contextual and methodological factors. For instance, as a multidimensional psychological construct, self-efficacy is simultaneously influenced by factors such as emotional regulation, stress perception, and social support (21), which may partially dilute the independent contribution of disease duration in the model. Furthermore, our sample consisted of community-based patients from primary care settings with relatively mild conditions (53). Given their lower baseline disease burden, the incremental benefit of prolonged disease duration on self-efficacy may be less pronounced. Nevertheless, even a small coefficient may represent meaningful psychological evolution within the long-term adaptation framework of chronic disease management. In other words, this small yet significant moderating effect reflects a slow but persistently accumulating reinforcement process, consistent with the “gradual consolidation” mechanism described in the Disease Adaptation Theory (34).

However, the observed discrepancies between our findings and the “long-term management fatigue” phenomenon reported in prior studies may be attributable to several factors: first, measurement and sampling particularities might have influenced the outcomes. Our multidimensional health literacy instrument—notably its inclusion of distributed health literacy emphasizing social resource mobilization capacities (18) —more authentically captures patients' ability to leverage familial care and community resources. For instance, long-duration patients with advanced health literacy may possess well-established medical-social support networks (20, 54), providing sustained behavioral reinforcement that maintains or enhances self-efficacy. Second, site-specific sampling characteristics warrant consideration. Whereas, previous studies on critically ill patients demonstrate that high treatment intensity and complex care needs exacerbate management fatigue and erode self-efficacy (55), our investigation focused on primary healthcare institutions predominantly serving patients with mild conditions (53). The relatively lower burden of long-term disease management in this population might have attenuated overt manifestations of management fatigue at the data level. Third, Conservation of Resources theory posits that when health literacy-derived management strategies form stable resource reservoirs, their automated implementation reduces cognitive load, thereby counteracting fatigue-inducing pressures (56). Furthermore, heterogeneous adaptation capacities mean not all long-duration patients experience efficacy belief deterioration—some develop psychological resilience through continuous learning and experiential accumulation (57). Fourth, the observed positive moderating effect of disease duration may stem from patients' progressive internalization of health literacy-constructed illness cognition into stable psychological resources. This cognitive reframing process reconfigures disease management as a life routine rather than an extraneous burden, potentially neutralizing fatigue's adverse impacts (58).

### Limitations

While this study systematically investigated the mechanism through which health literacy influences self-management behaviors via self-efficacy and revealed disease duration's moderating effects, several limitations warrant consideration. First, the exclusive recruitment of participants from primary healthcare institutions in Ouhai District, Wenzhou City—despite employing stratified sampling to enhance representativeness—limits generalizability given substantial regional disparities in healthcare resource allocation, chronic disease management protocols, and sociocultural contexts across China. These findings require further validation in broader populations. Second, reliance on self-reported questionnaires introduces potential biases. Although standardized protocols mitigated measurement errors, single-item assessment of disease duration remains vulnerable to recall bias, while evaluations of self-efficacy and self-management behaviors might be confounded by social desirability effects. Finally, the cross-sectional design precludes definitive causal inferences regarding temporal relationships among health literacy, self-efficacy, and self-management behaviors. Future longitudinal studies should establish causal pathways through repeated measurements and time-lagged analyses.

## Conclusions

Based on social cognitive theory and disease adaptation theory, this study constructs a mediating model in which health literacy influences self-management behaviors in patients with chronic diseases through self-efficacy. It systematically examines the mediating transformation mechanism of the “capability-belief-behavior” pathway within the four-dimensional health literacy framework and the moderating role of disease duration. This approach transcends the traditional individual capability limitations of health literacy and reconceptualizes disease duration characteristics from temporal variables to moderating factors. The research first confirms that self-efficacy plays a partial mediating role between health literacy and self-management behaviors, demonstrating that patients' confidence in their own capabilities serves as a crucial bridge connecting health literacy to the transformation of self-management behaviors. Furthermore, disease duration positively moderates the impact of health literacy on self-efficacy. Specifically, as disease duration increases, the indirect effect of health literacy on self-management behaviors through self-efficacy becomes significantly stronger. This reveals that the interplay between health literacy and self-efficacy is not static but dynamically evolves throughout the disease adaptation process, underscoring the critical importance of temporal dimensions in chronic disease management. At the practical level, this study establishes a theoretical foundation for developing precision intervention strategies tailored to different stages of illness progression.

## Data Availability

The raw data supporting the conclusions of this article will be made available by the authors, without undue reservation.
