# Supplementary material for: Mechanism of health literacy impact on self-management behaviors in patients with chronic disease: a self-efficacy mediated model moderated by disease duration
Source: Front Public Health. 2025 Nov 10;13:1673723. doi: 10.3389/fpubh.2025.1673723 (PMC12640832; doi:10.3389/fpubh.2025.1673723)
Supplement: Supplementary file 1 [file Presentation_1.pdf]

A Questionnaire on the Impact of Multidimensional Health Literacy on Self-Management Behavior of Patients with Chronic Diseases

Dear Respondents,

Greetings! We are researchers from Wenzhou Medical University focusing on health literacy. To understand your needs and challenges in managing chronic diseases, we are conducting a survey on the "Impact of Multidimensional Health Literacy on Self-Management Behaviors in Chronic Disease Patients" and we cordially invite your participation.

Your responses will be kept strictly confidential and used solely for research purposes. There are no right or wrong answers. We kindly ask you to respond based on your personal circumstances and genuine feelings. Thank you for your support and cooperation!

Wishing your health and happiness!

Survey Date: ____________________________

Survey Location: _________________________

Name of Investigator: _________________________

Questionnaire Code: _________________________

Respondent's Name: _________________________

School of Medical Humanities and Management, Wenzhou Medical University

July 2024

Part 1 Survey on Demographic Characteristics and Health Status

1. Have you been diagnosed with any of the following chronic diseases (You may choose more than one):

A. Hypertension, Coronary Heart Disease, Stroke, and Hyperlipidemia, which are cardiovascular and cerebrovascular conditions

B. Asthma, Chronic Obstructive Pulmonary Disease (COPD), and other chronic respiratory conditions

C. Diabetes or prediabetes (6.1 mmol/L $\leq$ fasting blood glucose $<$ 7.0 mmol/L)

D. Cancer

E. None of the above

2. How many years has it been since you first learned that you had a chronic illness:

A.0~2 years　　B.2~4 years　　C.4~6 years

D.6~8 years　　E.8~10 years　　F. More than 10 years

3. How would you rate your health status:

A. Very bad　　B. Bad　　C. Neutral　　D. Good　　E. Very good

4. Height and weight:

Height: __________m (to two decimal places)

Weight: __________Kg (to the nearest whole number)

5. Gender:　　A. Male　　B. Female

6. Date of birth: __________

7. Ethnicity:　　A. Han Chinese　　B. Ethnic minorities

8. Highest level of education attained:

A. Illiterate　　B. Primary school

C. Middle school　　D. High school

E. College　　F. Above college

9. Occupation:

A. Head of government agency, enterprise, or institution

B. Professional personnel (doctors, teachers, scientific researchers, etc.).

C. Office Staff

D. Company staff

E. Agricultural workers (farmers, forestry, herders, fishermen)

F. Freelancers

G. Unemployed

H. Retire

I. Other (please specify)

10. Marital status:

A. Unmarried

B. Married and residing with spouse

C. Married, but not currently living with spouse due to circumstances

D. Divorce

E. Widowhood

11. Residence:

A. City

B. Urban-rural fringe

C. Countryside

12. Number of household members：_______

13. Annual income for the past year (RMB):

A. (0, 40000]

B. (40000, 60000]

C. (60000, 80000]

D. (80000, 100000]

E. (100000, 150000]

F. More than 150000

G. I don't know

H. Refuse to answer

14. Annual household income for the past year (RMB):

A. (0, 50000]

B. (50000, 100000]

C. (100000, 150000]

D. (150000, 200000]

E. (200000, 250000]

F. (250000, 300000]

G. More than 300,000

H. I don't know

I. Refuse to answer

Part 2 Health Literacy Survey

1. Functional Health Literacy

When you read instructions or leaflets from hospitals or pharmacies, how do you agree or disagree about the following	Strongly disagree	Disagree	Not sure	Agree	Strongly agree
1. I found that there are some words that I can't read	5	4	3	2	1
2. It's difficult for me to extract the information I need regarding disease and its treatment	5	4	3	2	1
3. It's difficult for me to understand the information about the disease and its treatment	5	4	3	2	1

2. Communicative Health Literacy

**If you are diagnosed as having a disease and you have little information about the disease and its treatment, how do you agree or disagree about the following**	**Strongly disagree**	**Disagree**	**Not sure**	**Agree**	**Strongly agree**
1. I'm able to extract information about the disease and its treatment through interactions with doctors, family members, friends, and fellow patients	1	2	3	4	5
2. I'm able to understand the information expressed by doctors, family members, friends and fellow patients about the disease and its treatment	1	2	3	4	5
3. I can accurately convey my summaries and experiences with disease treatment to doctors, family, friends, fellow patients, and others	1	2	3	4	5
4. When I have questions, I will actively seek advice from doctors or professionals	1	2	3	4	5
5. In my interactions with others, I can accurately describe symptoms of diseases, medication usage, and diagnostic plans	1	2	3	4	5

3. Critical Health Literacy

**When you have been diagnosed with a disease and you can get information about the disease and its treatment, how do you agree or disagree about the following**	**Strongly disagree**	**Disagree**	**Not sure**	**Agree**	**Strongly agree**
1. I consider whether the information is applicable to me	1	2	3	4	5
2. I can consciously check that the information is valid and reliable	1	2	3	4	5
3. I can assess the reliability of the sources and channels that provide this information	1	2	3	4	5
4. I have the ability to evaluate the validity and scientific basis of this information	1	2	3	4	5
5. I collect information to make my healthcare decisions	1	2	3	4	5

4. Distributed Health Literacy

**When you have been diagnosed with a disease, please select according to your actual use of health resources available to you in the following table**	**Strongly disagree**	**Disagree**	**Not sure**	**Agree**	**Strongly agree**
1. The individuals around me possess the full capability to offer health care services or professional health management advice	1	2	3	4	5
2. The community or other relevant organizations frequently organize health-related activities, such as health seminars and forums, for us to participate in	1	2	3	4	5
3. My family and friends are well equipped to help me get and understand information about the disease and its treatment, and make informed decisions or give the right advice	1	2	3	4	5
4. I have access to health support organizations, such as patient groups, where I can obtain information about disease and its treatment, as well as receive advice and assistance from others	1	2	3	4	5

Part 3 Self-efficacy Survey

Completely Unconfident	Unconfident	Not sure	Confident	Completely confident
1. Confidence in keeping fatigue from interfering				
1	2	3	4	5
2. Confidence in keeping pain/physical discomfort from interfering				
1	2	3	4	5
3. Confidence in keeping emotional distress from interfering				
1	2	3	4	5
4. Confidence in keeping other symptoms from interfering (such as insomnia, lack of appetite, and lethargy)				
1	2	3	4	5
5. Confidence in reduce need to the doctor				
1	2	3	4	5
6. Confidence in reduce the illness effects				
1	2	3	4	5

Part 4 Survey of Patients' Self-Management Behaviors

**Please select the corresponding option**	**Strongly disagree**	**Disagree**	**Not sure**	**Agree**	**Strongly agree**
1. I consistently follow my doctor's recommendations and attend scheduled follow-up appointments without delay due to work or other reasons	1	2	3	4	5
2. I regularly monitor my symptoms and health status closely	1	2	3	4	5
3. I have a clear understanding of how to take my medications, including the dosage, potential side effects, and precautions	1	2	3	4	5
4. I do not take my medications with alcohol, juice, tea, coffee, or other beverages	1	2	3	4	5
5. I pay special attention to healthy eating habits, such as controlling oil, salt, and sugar intake, and maintaining a balanced diet	1	2	3	4	5
6. I will quit smoking and limit alcohol consumption to manage my condition	1	2	3	4	5
7. I usually maintain a regular lifestyle, going to bed early and waking up early each day to ensure ample sleep	1	2	3	4	5

8. I regularly create a rehabilitation exercise plan and purposefully select suitable forms of exercise, such as jogging, swimming, or tai chi	1	2	3	4	5
9. I adjust the type or intensity of my exercise based on changes in my condition	1	2	3	4	5
10. When my condition causes feelings of depression, I often seek comfort or support from family and friends	1	2	3	4	5
11. I often strive to cultivate positive emotions to help alleviate my condition	1	2	3	4	5
12. I frequently take the initiative to consult with doctors or professionals, or discuss with family and friends, when there are aspects of my treatment that I don't understand or want to learn more about	1	2	3	4	5
13. I do not avoid social activities in my daily life due to having a chronic disease	1	2	3	4	5
14. I often take the initiative to share my health status and management strategies with family, fellow patients, or others to gain their support and understanding	1	2	3	4	5
